# Calumenin-1 Interacts with Climp63 to Cooperatively Determine the Luminal Width and Distribution of Endoplasmic Reticulum Sheets

**DOI:** 10.1016/j.isci.2019.10.067

**Published:** 2019-11-02

**Authors:** Birong Shen, Pengli Zheng, Nannan Qian, Qingzhou Chen, Xin Zhou, Junjie Hu, Jianguo Chen, Junlin Teng

**Affiliations:** 1 Key Laboratory of Cell Proliferation and Differentiation of the Ministry of Education, State Key Laboratory of Membrane Biology, College of Life Sciences, Peking University, Beijing 100871, China; 2 Center for Quantitative Biology, Peking University, Beijing 100871, China; 3 College of Life Sciences, Jiangsu Normal University, Xuzhou 221116, China; 4 National Laboratory of Biomacromolecules and CAS Center for Excellence in Biomacromolecules, Institute of Biophysics, Chinese Academy of Sciences, Beijing 100101, China; 5 Department of Genetics and Cell Biology, College of Life Sciences, Nankai University and Tianjin Key Laboratory of Protein Sciences, Tianjin 300071, China

**Keywords:** Cell Biology, Membrane Architecture, Molecular Biology, Molecular Interaction, Organizational Aspects of Cell Biology

## Abstract

The ER is composed of distinct structures like tubules, matrices, and sheets, all of which are important for its various functions. However, how these distinct ER structures, especially the perinuclear ER sheets, are formed remains unclear. We report here that the ER membrane protein Climp63 and the ER luminal protein calumenin-1 (Calu1) collaboratively maintain ER sheet morphology. We show that the luminal length of Climp63 is positively correlated with the luminal width of ER sheets. Moreover, the lumen-only mutant of Climp63 dominant-negatively narrows the lumen of ER sheets, demonstrating that Climp63 acts as an ER luminal bridge. We also reveal that Calu1 specifically interacts with Climp63 and antagonizes Climp63 in terms of both ER sheet distribution and luminal width. Together, our data provide insight into how the structure of ER sheets is maintained and regulated.

## Highlights

• Climp63 determines the luminal width of ER sheets
• ER luminal protein Calumenin-1 (Calu1) interacts with Climp63
• Knockout of Calu1 triggers ER sheet accumulation and wider sheet lumen
• Calu1 regulates ER sheet morphology in a Climp63-dependent manner

## Graphical Abstract

**Figure:**
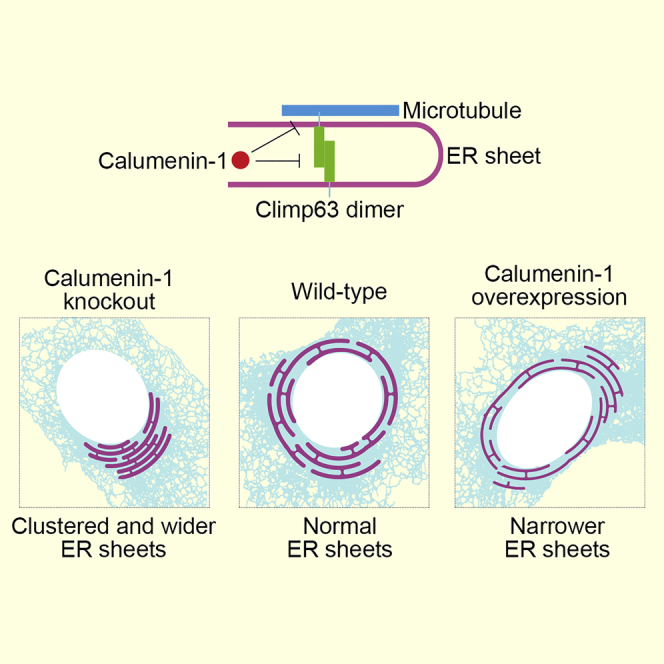

## Introduction

The ER is a continuous and highly dynamic membrane system distributing throughout the cytoplasm (Zhang and Hu, 2016). ER in mammalian cells is composed of a nuclear envelope and a peripheral network, which includes different structures like tubules, sheets, and matrices (Nixon-Abell et al., 2016, Schroeder et al., 2018, Shibata et al., 2010). ER subdomains exhibit distinctive functions. The ER sheets contain adherent ribosomes and are critical for luminal, membrane, and extracellular protein synthesis. The tubular ER is required for multiple processes including lipid synthesis (English and Voeltz, 2013, Jacquemyn et al., 2017) and signaling with other membranous organelles (Zhang and Hu, 2016, Zheng et al., 2018). However, how ER structures and networks, especially ER sheets, are formed and regulated remain unclear. It is also unknown whether ER luminal proteins contribute to the regulation of ER morphology.

The morphology of the tubular ER is formed and maintained primarily by membrane fusogen atlastins (Hu et al., 2009, Orso et al., 2009) and a subset of membrane curvature-stabilizing proteins, such as reticulons (Voeltz et al., 2006) and REEPs (Esteves et al., 2014, Park et al., 2010). Tubular ER-shaping proteins, such as reticulons, have also been proposed to stabilize the curvature edges of ER sheets, on the basis of observations that the reticulons also localize to the sheet edges and holes (Schroeder et al., 2018, Shibata et al., 2010). However, much less is known regarding the mechanism by which the ER sheets are segregated and regulated. Climp63, p180, and Kinectin have been identified as the most abundant integral ER membrane proteins with sheet-enriched localization and thus have been suggested to function in sheet formation (Shibata et al., 2010). Climp63 is a type II membrane protein that contains a single transmembrane segment and an extended coiled-coil domain in the ER lumen (Sandoz and van der Goot, 2015). The N terminus of Climp63, when dephosphorylated, interacts with microtubules (Vedrenne et al., 2005). Interestingly, p180 is also a microtubule-binding protein (Ogawa-Goto et al., 2007), and Kinectin was identified as a major binding partner of microtubule motor Kinesin (Toyoshima et al., 1992). Therefore, whether and how ER-microtubule interaction regulates ER sheet morphology will be intriguing to study. In neurons, the phosphorylation of Climp63 controls dendritic branching and cargo mobility via regulating ER complexity (Cui-Wang et al., 2012). It has been shown that knockdown of Climp63 led to narrower ER sheet lumen (Shibata et al., 2010), and Climp63 is thus considered as an ER luminal bridge. However, no other direct evidence has been provided regarding the bridging function of Climp63, and it is also unknown whether other proteins are involved.

Calumenin-1 (Calu1), belonging to CREC (acronym for Cab45, reticulocalbin, ERC-45, and calumenin) family, is a highly conserved ER luminal protein that can translocate into the ER lumen through the N-terminal signal peptide (Sahoo and Kim, 2010). CREC family proteins play important roles in various physiological processes, such as cell proliferation, cell migration, apoptosis, protein folding, calcium signaling, and intracellular transport (Chen et al., 2016, Deng et al., 2018, Honore, 2009, Wang et al., 2015). Calu1 contains multiple EF-hand domains and can interact with RyR2 and SERCA2 to regulate Ca^2+^ homeostasis in the sarcoplasmic reticulum in mouse heart (Sahoo et al., 2009). Knockdown of Calu1 increases γ-carboxylase activity, which in turn regulates the biogenesis of vitamin K-dependent proteins (Wajih et al., 2008). Calu1 acts as an ER stress chaperon in cardiomyocyte (Wang et al., 2017), and its overexpression alleviates the ER-stress-induced apoptosis (Lee et al., 2013). In addition, a part of Calu1 is secreted into the extracellular space, where it serves as a factor inhibiting tumor cell migration by binding to extracellular matrix protein fibulin-1 and suppressing the downstream ERK1/2 signaling pathway (Wang et al., 2015). However, whether Calu1 or any other ER luminal proteins are involved in regulating the ER morphology has not been reported.

Here, we present direct evidence that Climp63 determines the luminal width of ER sheets. In addition, we reveal that Calu1, an ER luminal protein, associates with and counteracts Climp63 to collaboratively regulate ER sheet morphology.

## Results

### Climp63 Determines the Luminal Width of ER Sheets

**Figure 1:**
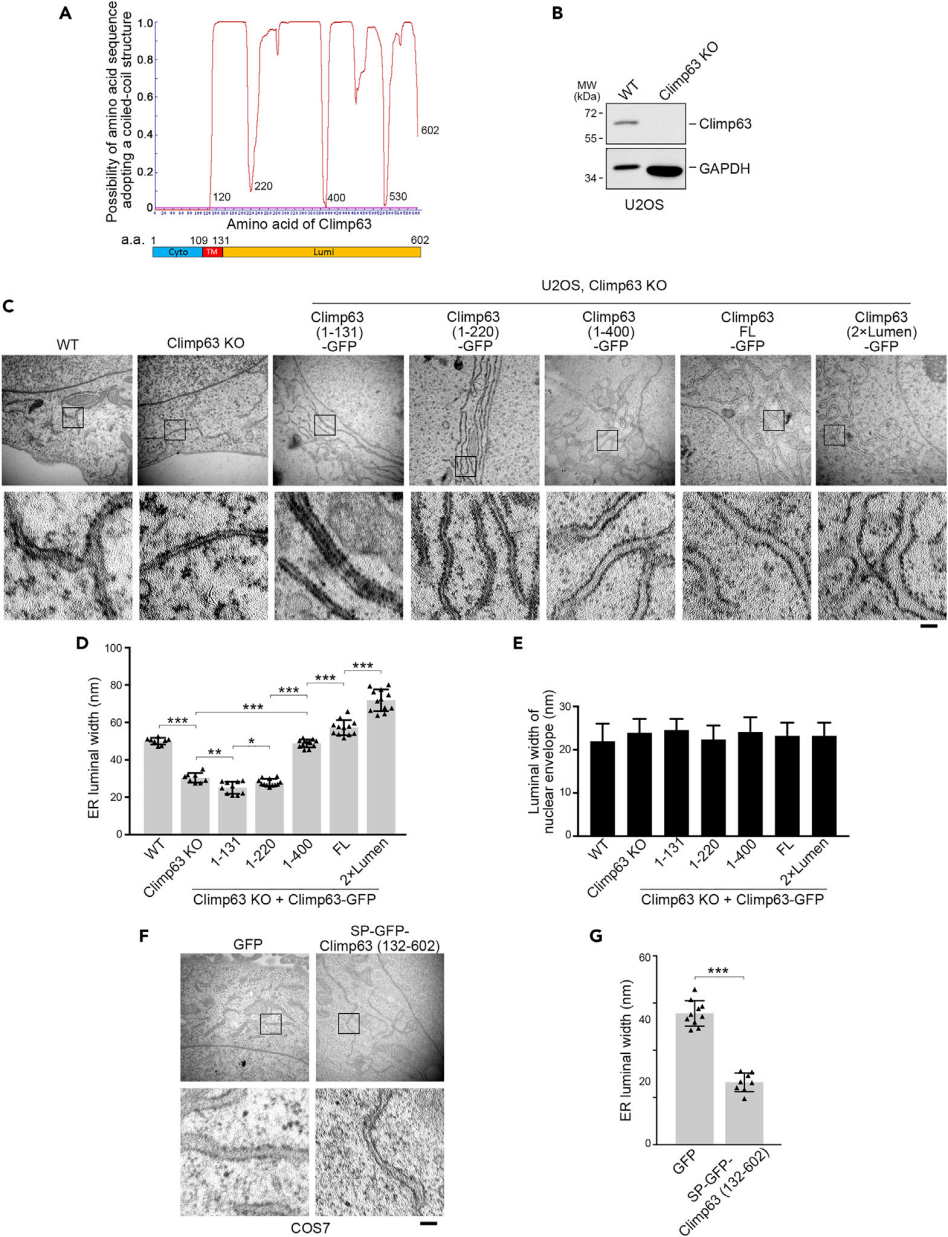
Climp63 Determines the Luminal Width of ER Sheets (A) Structure prediction of full-length Climp63 by coiled-coil domain prediction software tool LOGICOIL. It reveals that four coiled coils are formed by the luminal segment of Climp63. The red line stands for the Marcoil confidence level. Diagram depicting Climp63 domains is shown at the bottom. Numbers indicate amino acids (a.a.) of Climp63. Cyto, cytoplasmic domain; TM, transmembrane domain; Lumi, luminal domain. (B) Cell lysates from wild-type (WT) and Climp63 knockout (KO) cells were immunoblotted with anti-Climp63 antibody. GAPDH serves as a loading control. (C) Representative electron microscopy images of ER in wild-type or Climp63-knockout U2OS cells transfected with the indicated Climp63 mutant plasmids. Boxed regions are magnified below. Scale bar: 100 nm. (D) Quantification of the ER profile sheet luminal widths for (C). Data represent mean ± SD. *p < 0.05, **p < 0.01, ***p < 0.001, determined by unpaired two-tailed Student's t tests. (E) Quantification of the luminal widths of nuclear envelope profile for (C). Data represent mean ± SD. (F) Electron microscopy images of the ER in COS7 cells with overexpressed GFP or SP-GFP-Climp63 (132–602). SP, signal peptide (of Calu1). The boxed regions are magnified below. Scale bar: 100 nm. (G) Quantification of the ER profile sheet luminal widths for (F). Data represent mean ± SD. ***p < 0.001, determined by unpaired two-tailed Student's t test.

Climp63 is composed of a 109-amino-acid cytoplasmic tail, a single transmembrane domain, and a large ER luminal segment. To explore the functional domain of Climp63, we analyzed its amino acid sequence using LOGICOIL (Vincent et al., 2013). The results predicted that Climp63 contains four coiled-coil structures (amino acids 120–220, 220–400, 400–530, 530–602) (Figure 1A). A previous study (Shibata et al., 2010) showed that knockdown of Climp63 led to narrower ER lumen. To confirm whether Climp63 determines the luminal width of ER sheets, we generated Climp63-knockout U2OS cells by CRISPR/Cas9 (Figure 1B) and introduced Climp63 mutants with different luminal domain lengths (amino acids 1–131, 1–220, 1–400, full length, and 2×lumen) back into these cells to observe the sheet luminal widths by electron microscopy. Knockout of Climp63 led to much narrower sheet lumen (∼30 nm compared with ∼50 nm in wild-type U2OS cells) (Figures 1C and 1D), which was consistent with the previous report (Shibata et al., 2010). Interestingly, the lengths of the Climp63 luminal domains were positively correlated with ER sheet luminal widths: the shortest mutant Climp63-(1–131) showed the narrowest lumen, and the longest mutant Climp63-(2×lumen) showed the widest lumen (Figures 1C and 1D). As a control, the luminal width of the nuclear envelope showed no difference among mutants (Figure 1E), suggesting a specific regulation of Climp63 on ER sheets. These results strongly reveal that Climp63 determines the luminal width of ER sheets.

### Climp63 Self-Association through Its Luminal Domain Is Required for Determining the Luminal Width of ER Sheets

It has been hypothesized that Climp63 forms anti-parallel dimer to bridge the ER lumen (Shibata et al., 2009). To confirm whether dimerization of Climp63 luminal domain is responsible for determining the luminal width of ER sheets, we constructed a lumen-only mutant of Climp63 (amino acids 132–602) and localized it to the ER with an N-terminal signal peptide (Figure S1A). We reasoned that, if dimerization of Climp63 through the luminal coiled-coil regions is indispensable for its luminal bridge function, then overexpression of this mutant would dominant-negatively disrupt the normal function of the endogenous transmembrane Climp63, leading to narrower sheet luminal widths. Indeed, overexpression of this lumen-only mutant of Climp63 in COS7 cells resulted in much narrower ER sheet luminal widths (∼35 nm compared with ∼50 nm in control) (Figures 1F and 1G), indicating that dimerization through luminal coiled-coil domains is required for Climp63 to determine the luminal width of ER sheets.

### Calu1 Interacts with Climp63 and Regulates ER Sheet Luminal Width through Climp63

**Figure 2:**
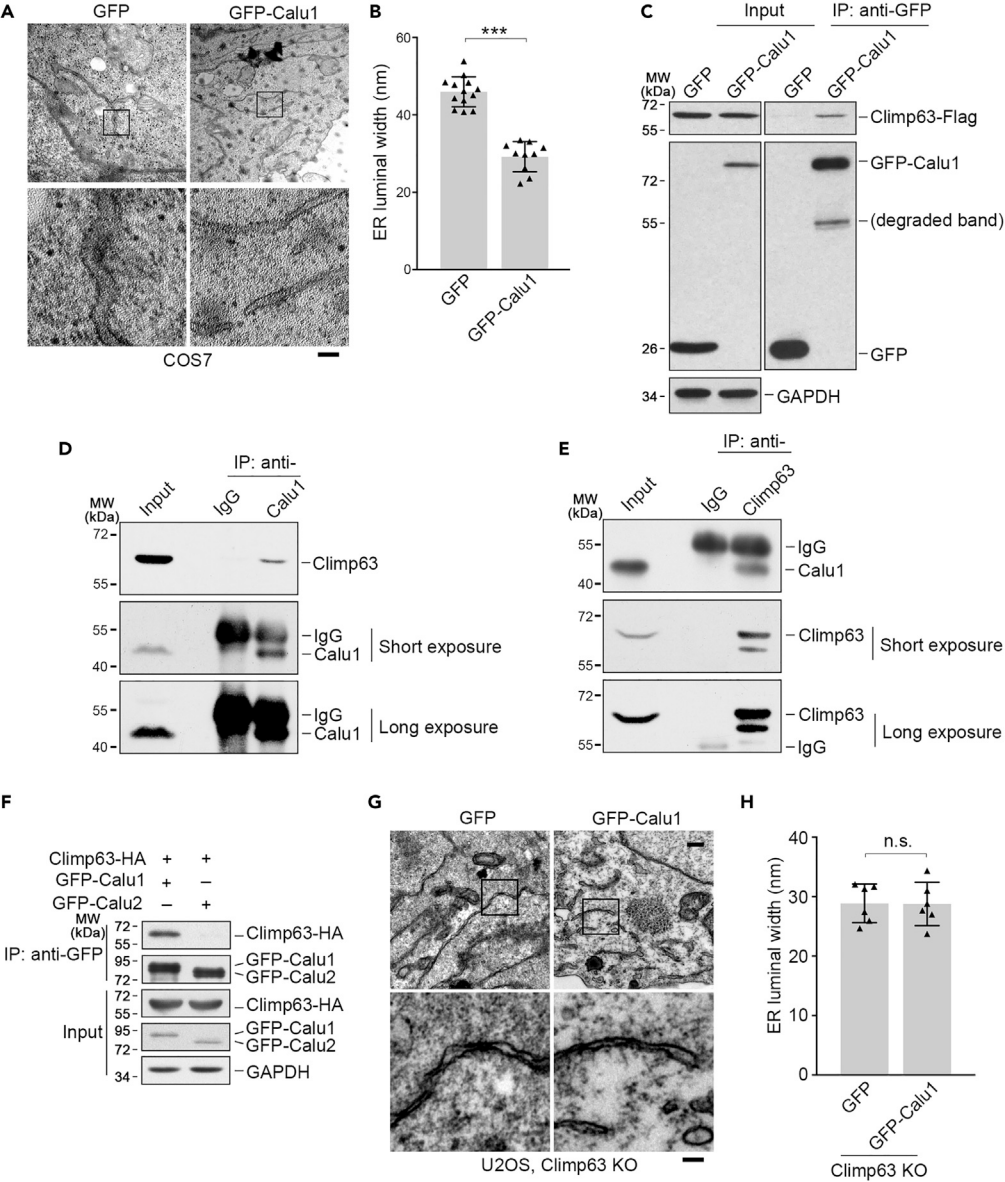
Calu1 Interacts with Climp63 and Regulates ER Sheet Luminal Width through Climp63 (A) Representative electron microscopy images of the ER sheets of COS7 cells with overexpressed GFP or GFP-Calu1. Boxed regions are magnified below. Scale bar: 100 nm. (B) Quantification of the ER profile sheet luminal widths for (A). Data represent mean ± SD. ***p < 0.001, determined by unpaired two-tailed Student's t test. (C) Immunoprecipitation (IP) assays of Climp63-Flag by overexpressed GFP or GFP-Calu1 in HEK293T cells. Cell extracts of Climp63-HA were mixed with cell extracts of GFP or GFP-Calu1 and then used for immunoprecipitation with anti-GFP antibody. The precipitates were immunoblotted with anti-GFP, anti-HA, and anti-GAPDH antibodies. GAPDH serves as a loading control. (D and E) Reciprocal endogenous immunoprecipitation assays with anti-Calu1 (D) or anti-Climp63 (E) antibodies in HEK293T cells. Normal IgG was used as a negative control. (F) Immunoprecipitation assays of Climp63-HA by GFP-Calu1 or GFP-Calu2 with anti-GFP antibody in HEK293T cells. (G) Representative electron microscopy images of the ER sheets of Climp63-knockout U2OS cells with overexpressed GFP or GFP-Calu1. Boxed regions are magnified below. Scale bar: 100 nm. (H) Quantification of the ER profile sheet luminal widths for (G). Data represent mean ± SD. n.s., not significant, determined by unpaired two-tailed Student's t test. See also Figures S1, S4, and S5.

We next sought to examine how Climp63 is regulated. Our laboratory has been studying the physiological roles of the proteins in the CREC protein family (Chen et al., 2016, Feng et al., 2013, Wang et al., 2015), and we found that the luminal width of ER sheets in COS7 cells overexpressing Calu1 (Figure S1B) was significantly narrower (∼30 nm compared with ∼50 nm in control) when analyzing the ER morphology using electron microscopy (Figures 2A and 2B). As Climp63 is the only reported protein to support ER luminal width, we determined to examine whether Climp63 interacts with Calu1. Immunoprecipitation assay using exogenously overexpressed proteins showed that Climp63 can be pulled down by GFP-Calu1 (Figure 2C). Furthermore, reciprocal immunoprecipitations confirmed that endogenous Calu1 associated with endogenous Climp63 (Figures 2D and 2E). This interaction was specific because Calu2, another major isoform of the *CALU* gene (Feng et al., 2013), did not associate with Climp63 (Figure 2F). In addition, immunoprecipitation assays between Calu1 and three other ER-shaping proteins, Kinectin (KTN1), p180, and Atlastin (Atl2), showed that Calu1 did not interact with any of these proteins (Figure S1C), suggesting a specific association between Calu1 and Climp63.

To examine whether narrower ER lumen induced by overexpression of Calu1 was dependent on Climp63, we overexpressed Calu1 in Climp63-knockout U2OS cells and analyzed the ER sheet morphology using electron microscopy. Overexpression of GFP-Calu1 in the Climp63-knockout U2OS cells showed indistinguishable ER luminal width compared with empty GFP transfected cells (Figures 2G and 2H), indicating that Climp63 is required for Calu1 to narrow ER lumen.

### Calu1 Regulates ER Sheet Distribution in a Climp63-Dependent Manner

**Figure 3:**
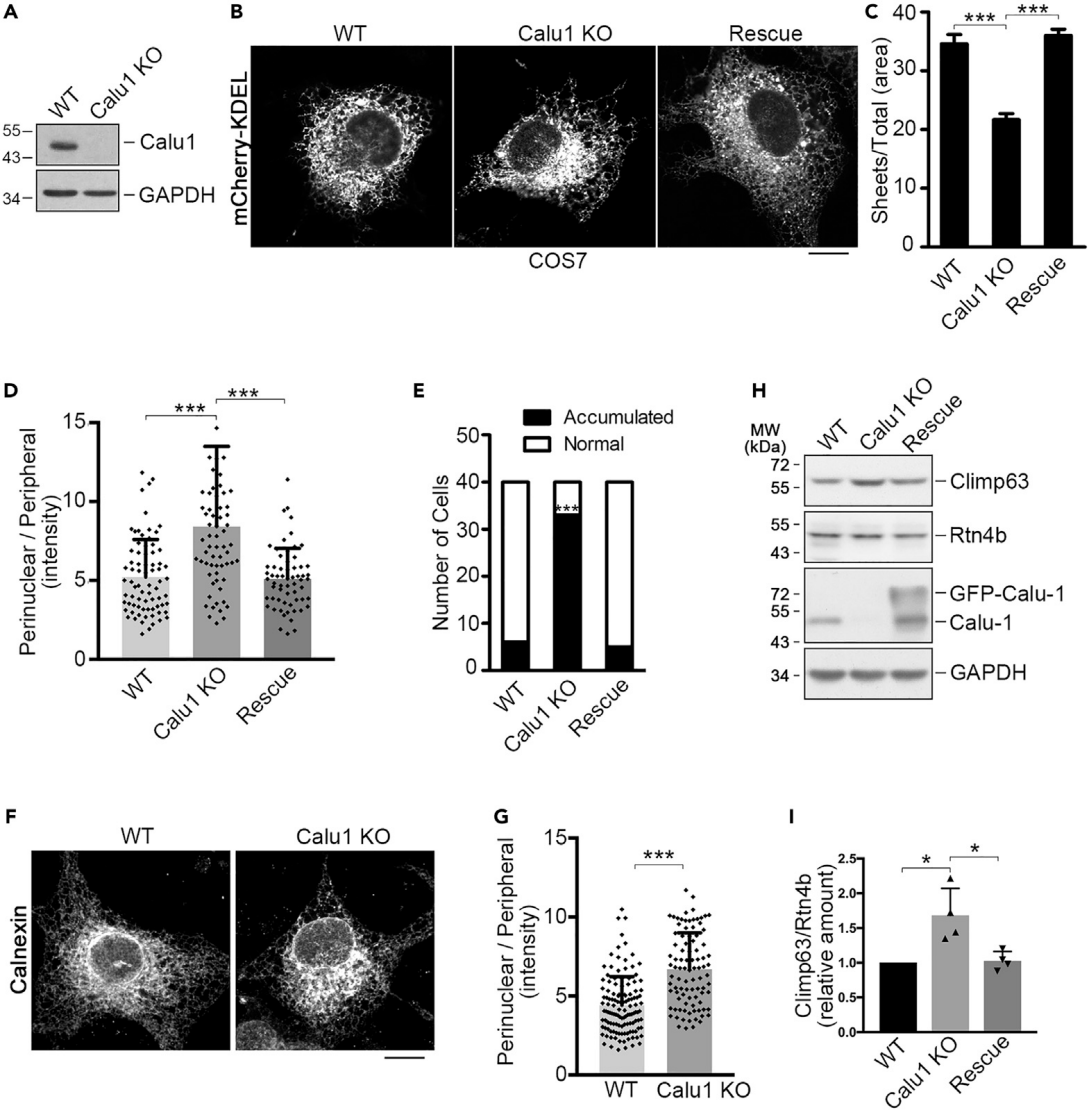
Loss of Calu1 Causes ER Sheet Accumulation (A) Western blotting of wild-type (WT) and Calu1-knockout (KO) COS7 cells by anti-Calu1 antibody. GAPDH serves as a loading control. (B) Representative confocal microscope images of wild-type, Calu1-knockout, and Calu1-knockout COS7 cells transfected with GFP-Calu1 plasmid (rescue). All cells are expressing mCherry-KDEL as an ER marker. Scale bar: 10 μm. (C) Quantification of relative distribution area of ER sheets to total ER of cells as in (B). Data represent mean ± SD. ***p < 0.001, determined by unpaired two-tailed Student's t tests. (D) Quantification of the relative average intensities of the perinuclear ER to peripheral ER in cells labeled by mCherry-KDEL as in (B). Data represent mean ± SD. ***p < 0.001, determined by unpaired two-tailed Student's t tests. (E) Quantification of the number of cells with juxtanuclear accumulation of ER as in (B). Data represent mean ± SD. ***p < 0.001, determined by unpaired two-tailed Student's t test. (F) Representative immunofluorescence images of wild-type and Calu1-knockout COS7 cells labeled with anti-Calnexin antibody. Scale bar: 10 μm. (G) Quantification of relative average intensities of the perinuclear ER to peripheral ER in cells labeled by endogenous Calnexin as in (F). Data represent mean ± SD. ***p < 0.001, determined by unpaired two-tailed Student's t test. (H) Western blotting of wild-type, Calu1-knockout COS7 cells, and Calu1-knockout COS7 cells restored with GFP-Calu1 (rescue) by anti-Climp63, anti-Rtn4 (Reticulon, Rtn4b), and anti-Calu1 antibodies. GAPDH serves as a loading control. Note that the band for Calu1 in the third lane is a degraded band. (I) Quantification of relative band intensities between Climp63 and Rtn4b as in (H). Data represent mean ± SD. *p < 0.05, determined by unpaired two-tailed Student's t tests. See also Figure S2.

We then prepared Calu1-knockout COS7 cells using the CRISPR/Cas9 approach (Figure 3A) and performed electron microscopy to examine whether knockout of Calu1 would affect ER sheet morphology. Calu1 knockout led to slightly wider ER sheet lumen (Figures S2A and S2B). Interestingly, we noticed that the ER sheets in Calu1 knockout cells seemed to be highly clustered (Figure S2A), so we set out to explore whether Calu1 affects the ER distribution using a luminal ER marker mCherry-KDEL (Zheng et al., 2018). Consistent with the electron microscopy results, knockout of Calu1 strongly disrupted the ER morphology, causing severe juxtanuclear accumulation of the ER (Figures 3B–3E), and restoration of Calu1 rescued the phenotype (Figures 3C–3E). To confirm these findings, we labeled Calnexin as an endogenous ER luminal protein marker in the Calu1 knockout cells. Consistent with the results shown by mCherry-KDEL (Figures 3B–3E), Calnexin labeling also showed accumulation of ER sheets in the perinuclear region (Figures 3F and 3G). As microtubules were reported to play critical roles in shaping the ER (Friedman et al., 2010, Waterman-Storer and Salmon, 1998), to investigate whether the accumulation of ER sheets affected the distribution of microtubule cytoskeleton, we labeled α-tubulin in both wild-type and Calu1 knockout cells but did not observe obvious alteration on the microtubule distribution (Figure S3A). In addition, the amount of ER sheets determined by the ratio of endogenous Climp63 (an ER sheet marker) to Rtn4b (a tubular ER marker) was elevated when Calu1 was knocked out (Figures 3H and 3I), indicating that not only are ER sheets accumulated, but also the amount is increased upon Calu1 deletion.

Calu1 was reported to be an ER stress chaperone, and its overexpression was demonstrated to alleviate the ER-stress-induced apoptosis (Lee et al., 2013, Wang et al., 2017). To test whether Calu1-knockout-induced ER sheets accumulation provokes ER stress, we examined the ER stress marker BiP, an ER heat shock protein 70 family member (Gething, 1999), in Calu1-knockout cells. Surprisingly, the BiP expression was markedly decreased when Calu1 was depleted (Figure S3B), suggesting the ER sheet accumulation does not induce ER stress.

**Figure 4:**
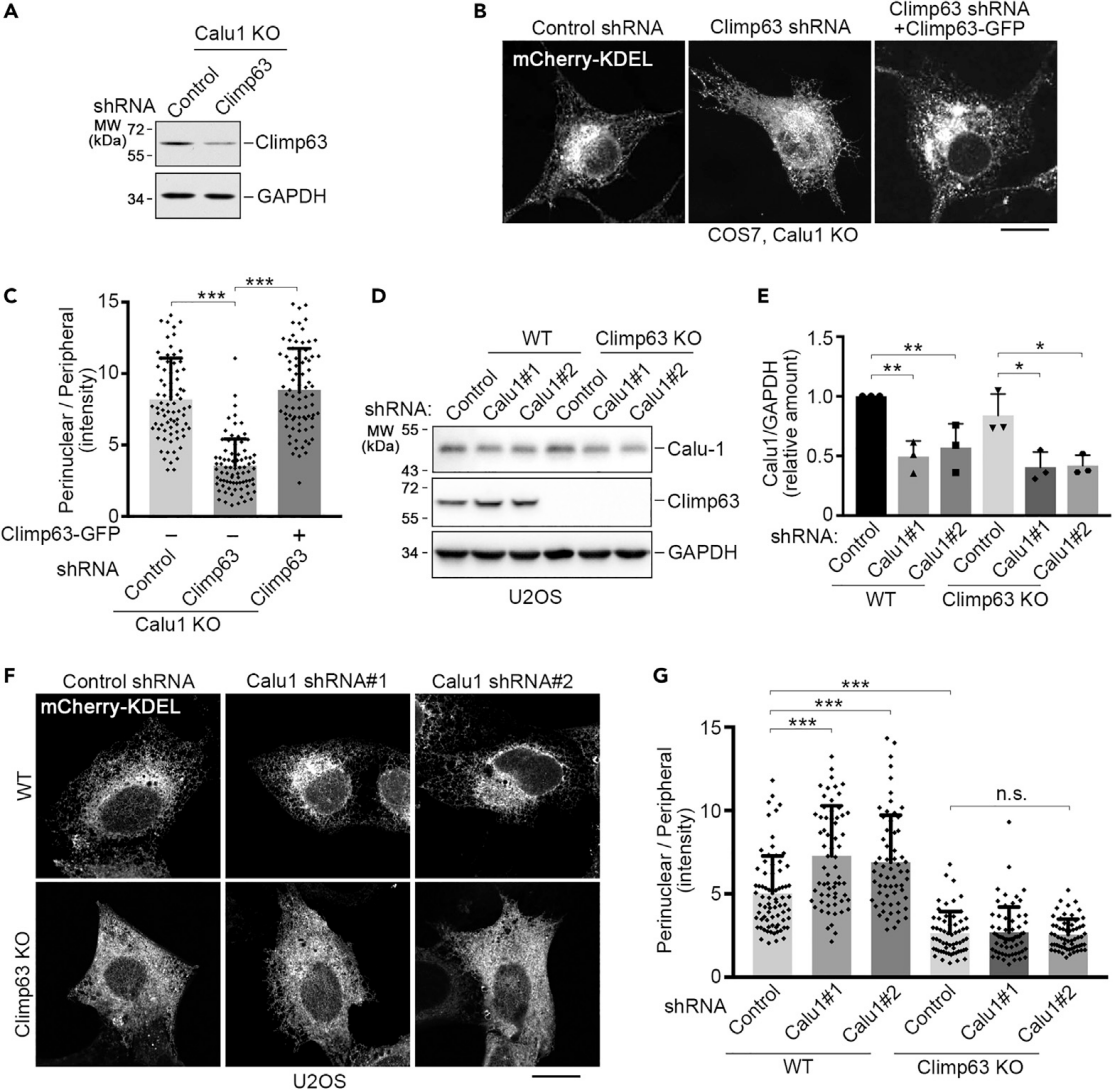
Calu1 Regulates ER Sheet Distribution in a Climp63-Dependent Manner (A) Western blotting of Calu1-knockout (KO) COS7 cells transfected with Climp63 shRNA and detected by anti-Climp63 antibody. GAPDH serves as a loading control. (B) Representative confocal microscope images of Calu1-knockout COS7 cells transfected with control shRNA, Climp63 shRNA, and Climp63 shRNA together with Climp63-GFP plasmid (Climp63 shRNA-resistant). All cells were expressing mCherry-KDEL as an ER marker. Scale bar: 10 μm. (C) Quantification of the relative average intensities of the perinuclear ER to peripheral ER in cells labeled by mCherry-KDEL as in (B). Calu1-knockout COS7 cells were transfected with control shRNA, Climp63 shRNA, or Climp63 shRNA and shRNA-resistant Climp63-GFP plasmid. Data represent mean ± SD. ***p < 0.001, determined by unpaired two-tailed Student's t tests. (D and E) Western blotting analysis and quantification of the knockdown efficiency of Calu1 in wild-type (WT) and Climp63-knockout U2OS cells. Data represent mean ± SD. *p < 0.05, **p < 0.01, determined by unpaired two-tailed Student's t tests. (F) Representative confocal microscope images of wild-type or Climp63-knockout U2OS cells transfected with mCherry-KDEL and the indicated shRNAs. Scale bar, 10 μm. (G) Quantification of the relative average intensities of the perinuclear ER to peripheral ER in cells labeled by mCherry-KDEL as in (F). Data represent mean ± SD, ***p < 0.001, n.s., not significant, determined by unpaired two-tailed Student's t tests. See also Figure S3.

To determine whether the ER accumulation is Climp63 dependent, we knocked down Climp63 in Calu1-knockout COS7 cells using shRNA (Figure 4A) and observed its effect on the juxtanuclear accumulation of ER. Consistent with a previous report (Shibata et al., 2010), knockdown of Climp63 caused dramatically dispersed ER sheets (Figures 4B and 4C), whereas the distribution of microtubule cytoskeleton did not seem to be affected (Figure S3C). Depletion of Climp63 efficiently and specifically reversed the juxtanuclear accumulation of ER induced by Calu1 knockout (Figures 4B and 4C), suggesting that Climp63 is required for Calu1 to modulate ER distribution. To further validate this, we knocked down Calu1 in Climp63-knockout U2OS cells (Figures 4D and 4E). Knockdown of Calu1 in wild-type U2OS cells induced dramatic juxtanuclear accumulation of ER; however, it failed to do so in Climp63-knockout U2OS cells (Figures 4F and 4G). Therefore, we conclude that Calu1 modulates ER distribution in a Climp63-dependent manner.

### Calu1 Binds to Climp63 *via* Its Amino Acids 74–138

We determined to map the interaction domain of Calu1 and Climp63. As only Calu1, but not Calu2, interacted with Climp63 (Figure 2F), and GFP-Calu2 also failed to rescue the accumulated ER sheets in Calu1 knockout cells (Figures S4A and S4B), we speculated that the different regions between Calu1 and Calu2, which are their exon4 regions (Wang et al., 2015), might be responsible for the interaction of Calu1 with Climp63. We constructed several Calu1 mutants (Figure S5A) and performed immunoprecipitation assays. As expected, Calu1 mutant lacking exon4 region (Calu1-Δ74-138) did not associate with Climp63 (Figure S5B). Consistently, this mutant failed to rescue the juxtanuclear accumulation of ER (Figures S5C and S5D). In contrast, Calu1-Δ20-73 (lacking exon3 region) associated with Climp63 and rescued the ER distribution abnormality as the full-length Calu1 (Figures S5B–S5D). Together, these data suggest that amino acids 74–138 of Calu1 are required for Calu1 to interact with Climp63 and, more importantly, are also required for Calu1 to regulate ER sheet distribution through Climp63.

### Calu1 Inhibits Climp63-Microtubule Binding but Does Not Affect Self-Association of Climp63

**Figure 5:**
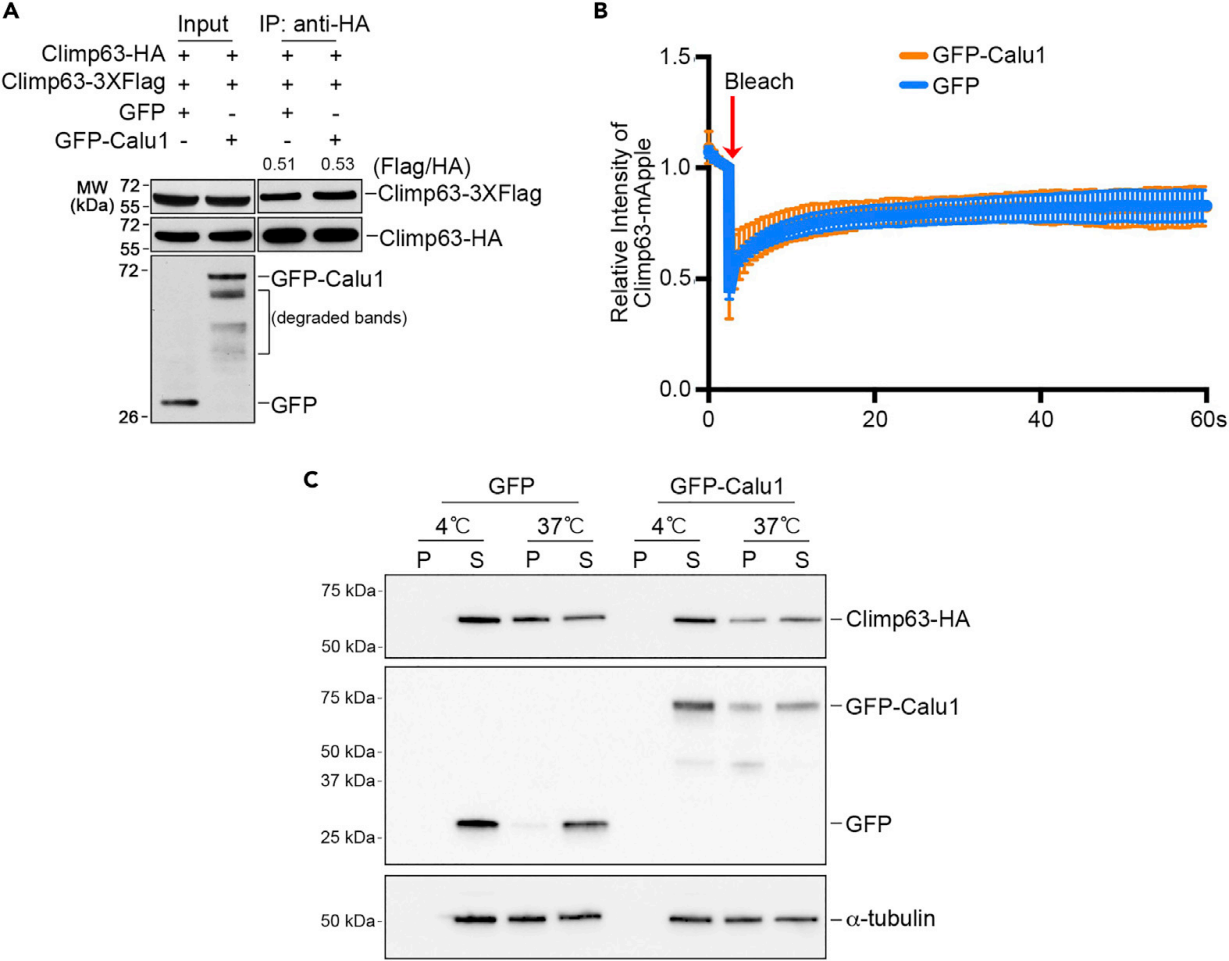
Calu1 Inhibits Climp63-Microtubule Binding Instead of Affecting Self-Association of Climp63 (A) Immunoprecipitation assays of Climp63-3×Flag by Climp63-HA using anti-HA antibody in HEK293T cells in the presence of GFP or GFP-Calu1. The number on top of the bands is the ratio between Climp63-3×Flag and Climp63-HA with GFP or GFP-Calu1 overexpression. (B) FRAP assay of COS7 cells with overexpressed Climp63-mApple and GFP or GFP-Calu1. The relative intensity of Climp63-mApple in the bleached area was recorded. Data shown are the average traces from 25 cells. (C) Microtubule co-sedimentation assay of HEK293T cells with overexpressed Climp63-HA and GFP or GFP-Calu1. At 37°C, less Climp63 exists in the pellet fraction (P) upon GFP-Calu1 overexpression (P% = 36%) compared with GFP overexpression (P% = 60%). Note that all Climp63 is in the supernatant fraction (S) at 4°C because microtubules are depolymerized at low temperature. P, pellet, microtubule fraction; S, supernatant, free tubulin fraction.

We next explored possible mechanisms of how Calu1 regulates ER morphology through Climp63. It was proposed that Climp63 exerts its luminal bridge function by forming anti-parallel dimers on opposing ER membranes (Shibata et al., 2009), and our data supported that dimerization through its luminal domain is important for Climp63 to regulate the ER luminal width (Figures 1F and 1G). Therefore, we tested whether Calu1 affects self-association/dimerization of Climp63. We first used immunoprecipitation assay to reveal that Climp63 can form a homodimer between Climp63-HA and Climp63-3×Flag. However, overexpression of GFP-Calu1 did not change the amount of Climp63-3×Flag pulled down by Climp63-HA (Figure 5A). Similarly, in a fluorescence recovery after photobleaching (FRAP) assay using Climp63-mApple, we did not observe any difference in the recovery rate between control and GFP-Calu1 overexpressed cells (Figure 5B). Therefore, it is unlikely that Calu1 affects self-association of Climp63.

Climp63 was reported to bind microtubules (Vedrenne et al., 2005). We determined to examine whether Calu1 affects the Climp63-microtubule association using the microtubule co-sedimentation assay. In control HEK293T cells expressing GFP and Climp63-HA (at 37°C), more Climp63 appeared in the pellet (microtubules) fraction (∼60%), suggesting that Climp63 associates with microtubules. However, in cells overexpressing GFP-Calu1 and Climp63-HA (at 37°C), only 36% of Climp63 appeared in the microtubule fraction (Figure 5C). Together, we speculate that Calu1 suppresses the microtubule-binding ability of Climp63.

## Discussion

The ER consists of a complex network, and disruption of ER morphology is closely linked to various human diseases, such as hereditary spastic paraplegia (Blackstone, 2012) and Alzheimer disease (He et al., 2004). Although the tubular ER-shaping proteins have been extensively studied, how ER sheets are formed and regulated are still unclear. Here, we show that ER transmembrane protein Climp63 and ER luminal protein Calu1 cooperatively maintain and regulate ER sheet morphology.

### Climp63 Acts as an ER Sheet Luminal Bridge

Although a previous model puts Climp63-bridged sheet lumen as one of the three possible mechanisms of ER sheet formation (Shibata et al., 2009), the speculation is based on indirect evidence, including the predicted molecular structure of Climp63 and the phenotype of Climp63 knockdown (Klopfenstein et al., 2001, Shibata et al., 2010). By showing that the luminal width correlates well with the luminal length of Climp63 (Figure 1), we provide solid evidence revealing that Climp63 determines the luminal width of ER sheets. We also show that the luminal coiled-coil region of Climp63 seems to be important for its luminal bridging function (Figures 1F and 1G), supporting the previous hypothesis that Climp63 molecules at the opposite ER membranes form anti-parallel dimer to support the ER lumen (Shibata et al., 2009, Shibata et al., 2010).

We notice that re-introduction of Climp63 mutant lacking a luminal domain (Climp63-1–131) results in an even narrower lumen compared with that in Climp63-knockout cells (Figures 1C and 1D). One possibility is that the luminal width of ER sheets is determined by a combination of Climp63-mediated luminal bridging and p180/Kinectin-mediated membrane scaffolding (Shibata et al., 2009). Therefore, the cytoplasmic domain of Climp63 may recruit p180 and Kinectin, leading to increased membrane scaffolding at the cytosolic side and a much narrower ER lumen.

Another interesting observation is that rescue of Climp63 knockdown by full-length Climp63 leads to a wider ER lumen compared with that in the wild-type cells (Figures 1C and 1D). One explanation is that, although Climp63 is among the most abundant proteins in the ER (Shibata et al., 2010), it is not saturated to support the lumen of all ER sheets, or only a portion of Climp63 is involved in the luminal bridging. Thus, overexpression of full-length Climp63 adds more bridges inside the ER and leads to a wider lumen. Another possibility is that the luminal width of ER sheets is not only determined by Climp63 in the lumen, but also involving a pressing force generated by p180/Kinectin from the cytosol side (Shibata et al., 2009). Therefore, increased amount of Climp63 will give more strength from the luminal bridge side and slightly increase luminal width.

### ER Luminal Proteins and ER Morphology

The proteins that have been reported to function in ER tubule and sheet formation or to balance ER tubules and sheets are all membrane-associated proteins (Hu et al., 2008, Hu et al., 2009, Park et al., 2010, Shibata et al., 2010). Our data reveal that Calu1, an ER luminal protein, collaborates with Climp63 in modulating ER sheet morphology. This finding broadens the existing knowledge and provides insights for studying ER morphology. Calu1 was reported to be an ER chaperon, which alleviated the ER stress-induced apoptosis in cardiomyocyte (Lee et al., 2013, Wang et al., 2017). To our surprise, although knockout of Calu1 triggered accumulation of ER distribution, it does not cause ER stress indicated by BiP (Figure S3B) in COS7 cells. These discrepancies may be due to cell type, or that the changed ER distribution alleviates ER stress.

### Mechanisms Underlying Regulation of Calu1 on Climp63

Our data show that not only Calu1 regulates the luminal width of ER, but its knockout also triggers juxtanuclear accumulation of ER, and both are Climp63 dependent. However, the causal relationships between these two phenotypes are unclear. How Calu1 regulates ER morphology via Climp63 is another open question. One possibility is that Calu1 might regulate the self-association of Climp63. Although we did not detect any effect of Calu1 on Climp63 self-association (Figures 5A and 5B), it is still possible that Calu1 could regulate the arrangement of Climp63. There is one scenario that only anti-parallel Climp63 dimers/polymers formed from opposing ER membranes can support the lumen (Shibata et al., 2009); however, the parallel dimers/polymers are dominant in the cells. Therefore, if Calu1 specifically inhibits the anti-parallel dimerization/polymerization of Climp63, which comprises only a minor proportion of Climp63 dimers/polymers, it would be difficult to detect differences with or without Calu1 by the method we used to test Climp63 dimerization (Figures 5A and 5B). Unfortunately, we could not think of a method to specifically detect anti-parallel dimerization of Climp63.

Another possibility, which we have validated, is that Calu1 affects the Climp63-microtubule binding. This possibility stems from the finding that the cytoplasmic domain of Climp63 was reported to contain microtubule-binding domains (Klopfenstein et al., 1998) and this microtubule binding is involved in regulating ER morphology (Vedrenne et al., 2005). Despite this result, we do not have a clear explanation how Calu1 regulates the Climp63-microtubule binding as a luminal protein. One possibility is that binding of Calu1 to Climp63 changes the structure of Climp63, which may also be the underlying mechanism of the ER sheet luminal width regulation. It is also worthwhile to mention that the expression of Climp63 was increased in Calu1-knockout cells (Figure 3H), which may also contribute to the ER phenotype. Therefore, Calu1 may regulate Climp63 in several different ways. Further studies are required to address questions like which contributes more to the phenotype and what are their relationships among different mechanisms.

### Limitation of the Study

This study did not investigate whether Climp63 self-association is parallel or anti-parallel. It remains unclear how Calu1 affects Climp63-microtubule binding as an ER luminal protein. We speculate that binding of Calu1 to Climp63 might change the conformation of Climp63.

## Methods

All methods can be found in the accompanying Transparent Methods supplemental file.

## Supplemental Information

Document S1. Transparent Methods and Figures S1–S5
