# Supplementary material for: Calumenin-1 Interacts with Climp63 to Cooperatively Determine the Luminal Width and Distribution of Endoplasmic Reticulum Sheets
Source: iScience. 2019 Nov 2;22:70–80. doi: 10.1016/j.isci.2019.10.067 (PMC6931119; doi:10.1016/j.isci.2019.10.067)
Supplement: Document S1. Transparent Methods and Figures S1–S5 [file mmc1.pdf]

ISCI, Volume *22*

Supplemental Information

Calumenin-1 Interacts with Climp63 to Cooperatively Determine the Luminal Width and Distribution of Endoplasmic Reticulum Sheets

Birong Shen, Pengli Zheng, Nannan Qian, Qingzhou Chen, Xin Zhou, Junjie Hu, Jianguo Chen, and Junlin Teng

Supplemental Information

Supplemental Figures

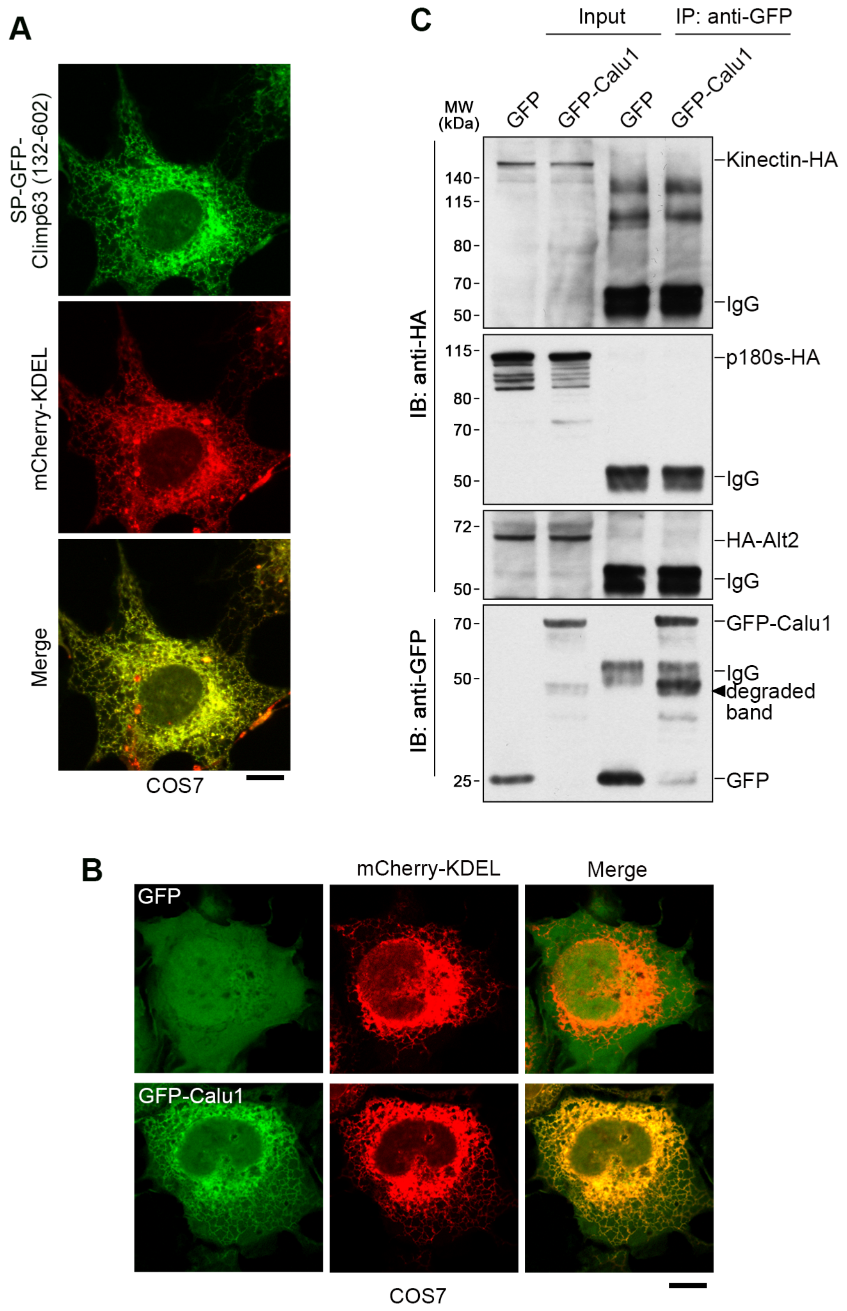

Figure S1. ER Localization of Climp63 Dominant-Negative Mutant and GFP-Calu1, and Calu1 Does Not Interact with KTN1, p180 or Atl2. Related to Figure 2

(A) Representative images of COS7 cells expressing SP-GFP-Climp63 (132-602) and an ER marker mCherry-KDEL. Scale bar: 10 μm.

(B) Representative images of COS7 cells expressing either GFP or GFP-Calu1 and an ER marker mCherry-KDEL. Scale bar: 10 μm.

(C) Immunoprecipitation (IP) assays of overexpressed KTN1-HA (Kinectin), p180s-HA (a short isoform of p180), or Atl2-HA (Atlastin) by overexpressed Calu1-GFP in HEK293T cells with anti-GFP antibody. The precipitates were immunoblotted (IB) with anti-GFP and anti-HA antibodies.

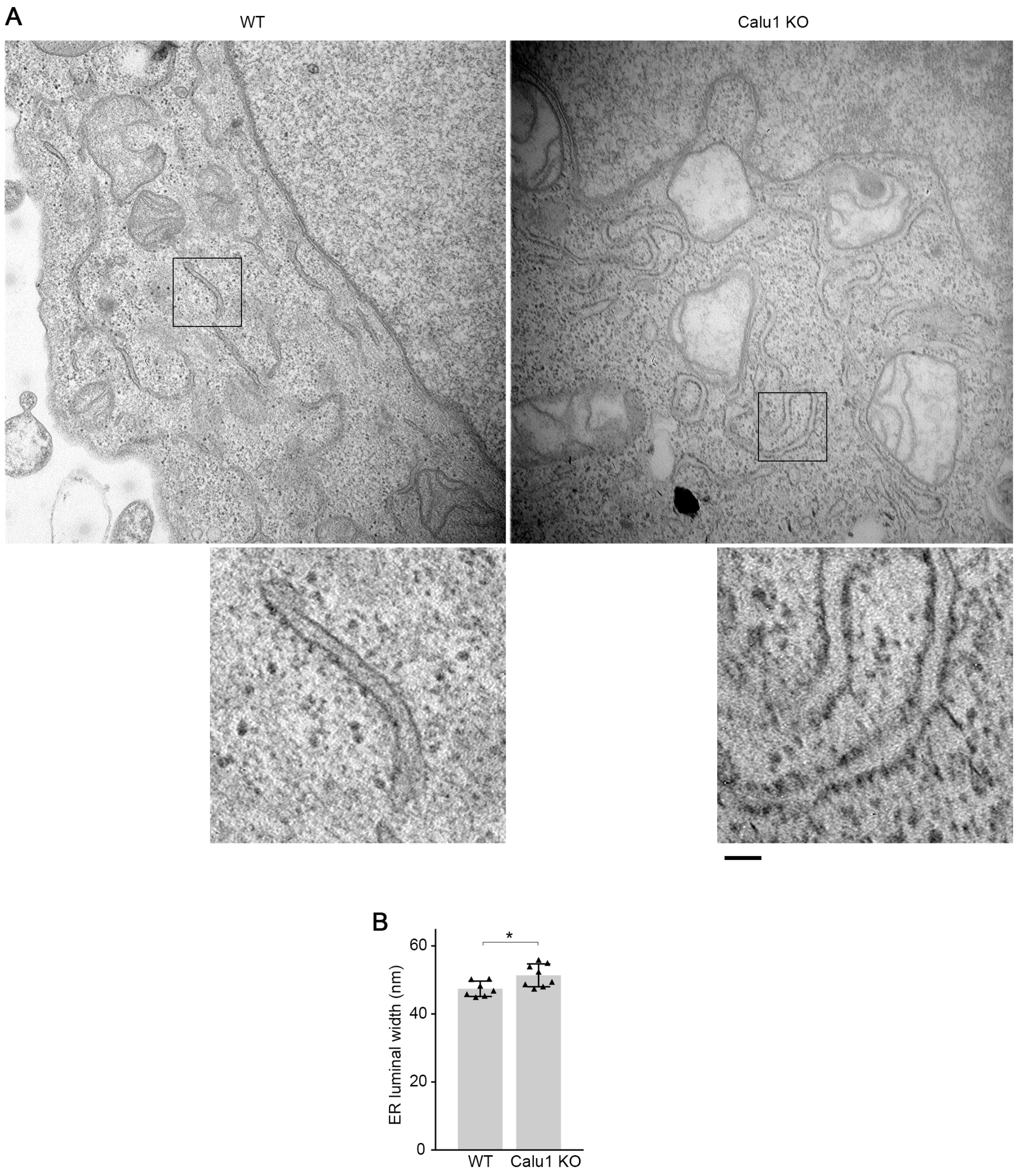

Figure S2. Calu1 Knockout Leads to Wider ER Sheet Luminal Width. Related to Figure 3

(A) Representative electron microscopy images of wild-type (WT) and Calu1-knockout (KO) COS7 cells. Boxed regions are magnified below. Scale bar: 100 nm.

(B) Quantification of the ER sheet luminal widths for (A). Data represent mean ± SD, *$p < 0.05$, determined by unpaired two-tailed Student's t-test.

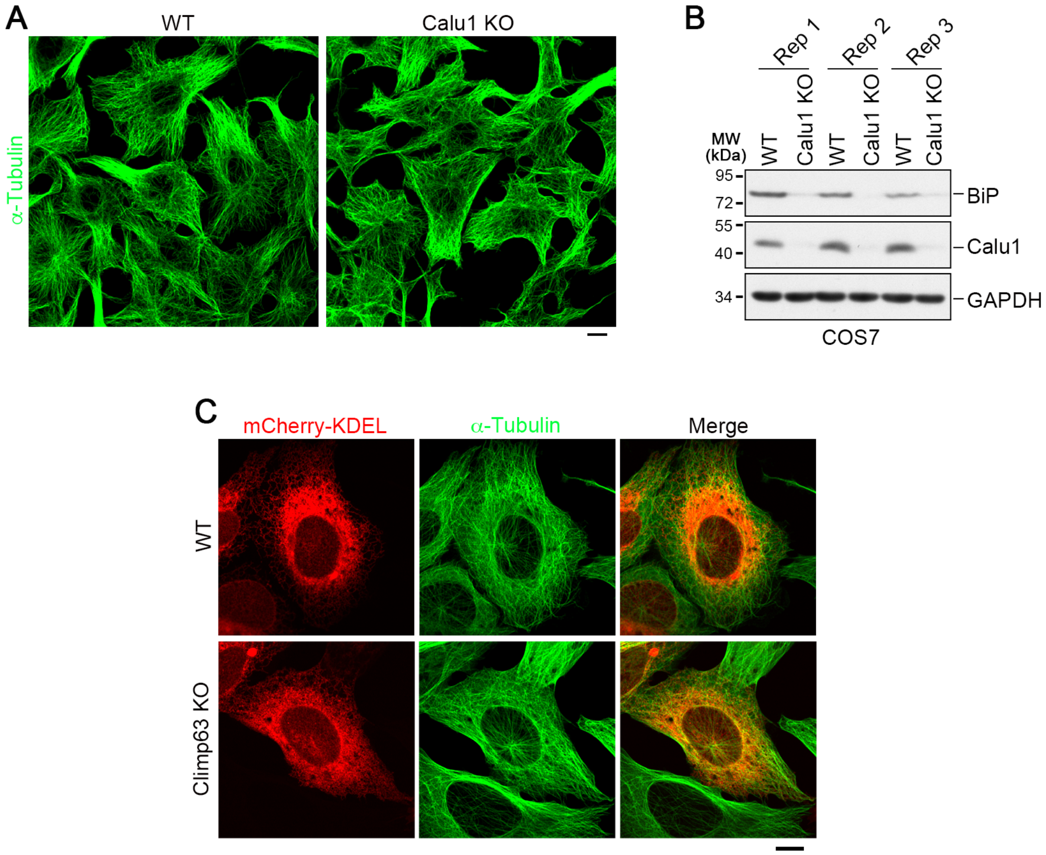

Figure S3. Knockout of Calu1 or Climp63 Does Not Affect Microtubule Distribution and Calu1 Deletion Decreased ER Stress. Related to Figure 4

(A) Representative immunofluorescence confocal microscopy images of wild-type (WT) and Calu1-knockout (KO) COS7 cells labeled with α-tubulin. Scale bar: 10 μm.

(B) Western blotting of BiP in wild-type and Calu1-knockout cells. Rep 1, 2, and 3 indicate three independent replications. GAPDH serves as a loading control.

(C) Representative immunofluorescence confocal microscopy images of wild-type and Climp63-knockout U2OS cells transfected with mCherry-KDEL and labeled with α-tubulin. Scale bar: 10 μm.

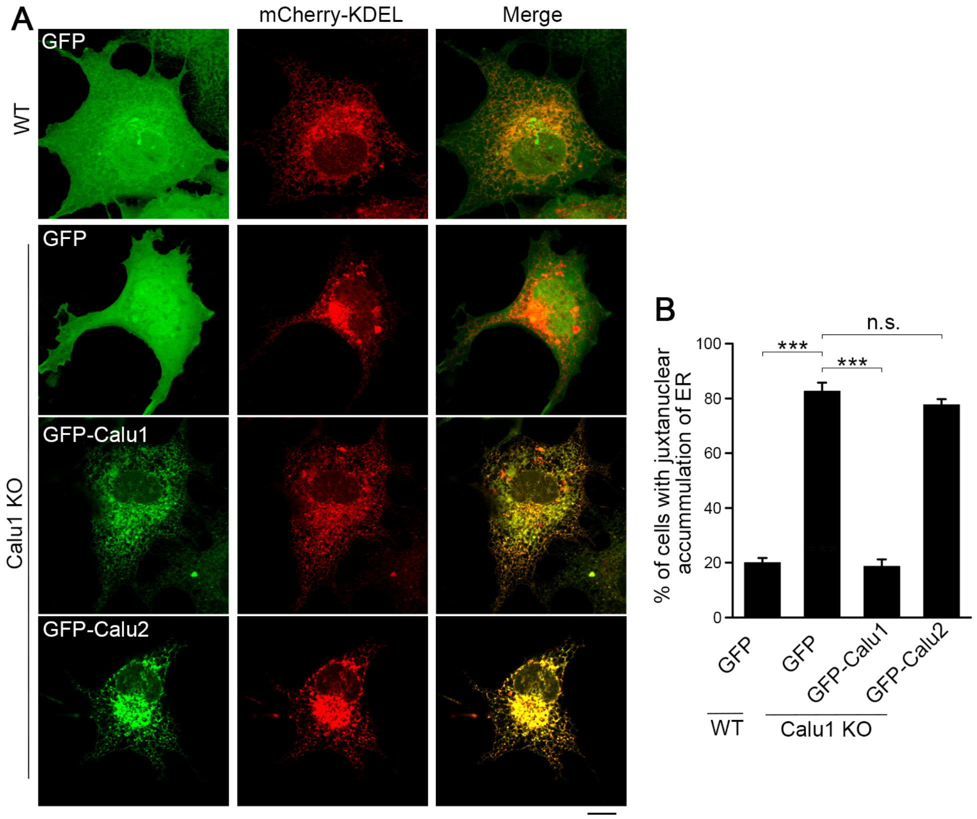

Figure S4. Restoration of Calu1 But Not Calu2 Rescued the ER Sheet Accumulation in Calu1-Knockout COS7 Cells. Related to Figure 2

(A) Representative images of wild-type (WT) or Calu1-knockout (KO) COS7 cells transfected with the indicated plasmids. Scale bar: 10 μm.

(B) Quantification of cells with juxtanuclear accumulation of ER in (A). Data represent mean ± SD, ***$p < 0.001$, n.s., not significant, determined by unpaired two-tailed Student's t-tests.

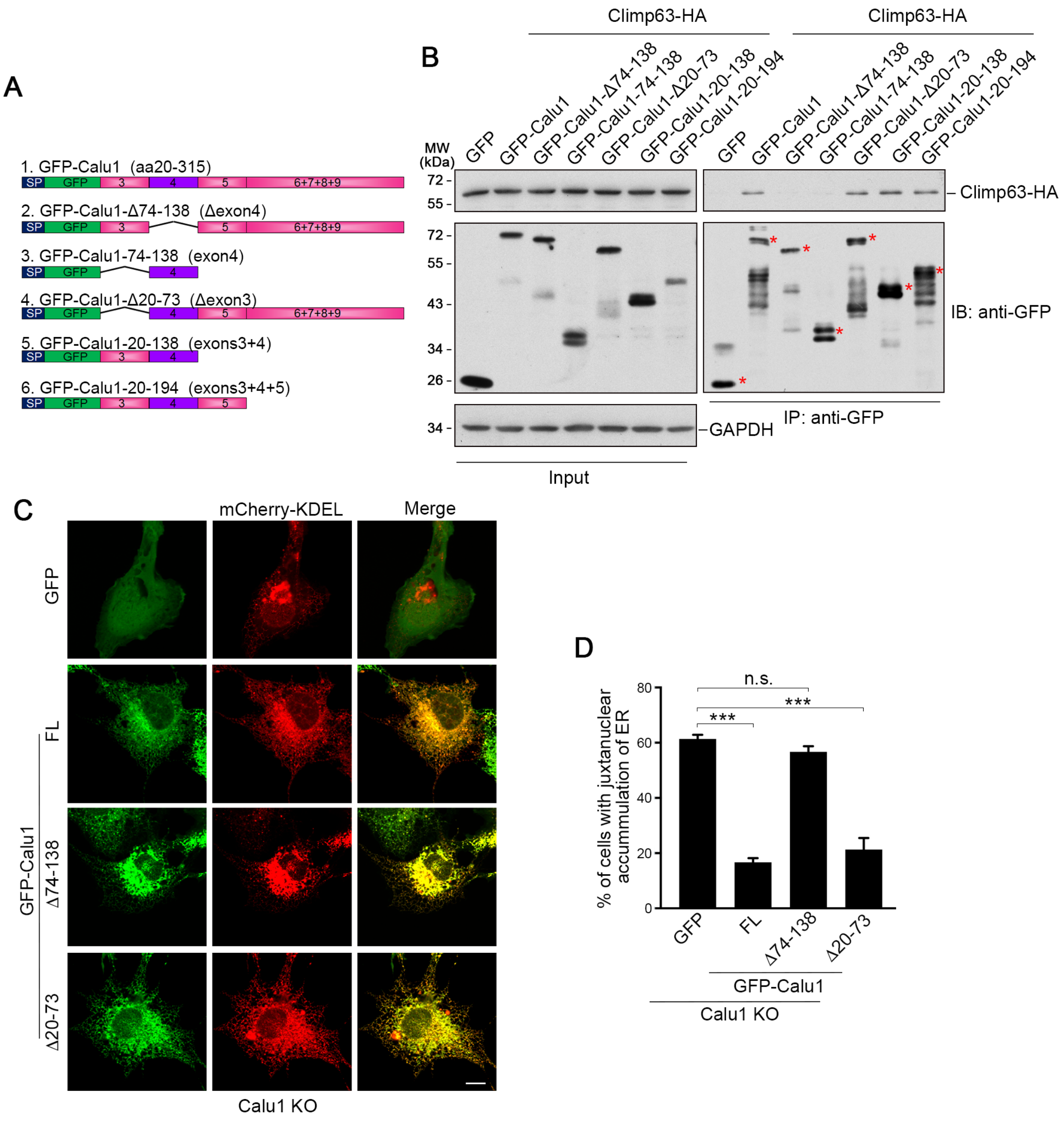

Figure S5. Calu1 Interacts with Climp63 *via* Amino Acids 74-138. Related to Figure 2

(A) Schematic illustration of Calu1 deletion mutants. The numbers inside the diagram indicate exons of CALU gene. SP, signal peptide of Calu1. The GFP sequence was inserted directly after the signal peptide.

(B) Immunoprecipitation (IP) assays of Climp63-HA by the indicated Calu1 deletion mutants overexpressed in HEK293T with anti-GFP antibody. The red asterisks indicate the expected bands for the immunoprecipitated Calu1 mutants. IB, immunoblotting.

(C-D) Calu1-Knockout (KO) COS7 cells were overexpressed with mCherry-KDEL and GFP or the indicated Calu1 mutants. Percentage of cells with juxtanuclear accumulation of ER was quantified. FL, full length. Data represent mean ± SD, ***$p < 0.001$, n.s., not significant, determined by unpaired two-tailed Student's t-tests.

Transparent Methods

Cell Culture and Transfection

HeLa (ATCC, CCL-2), HEK293T (ATCC, CRL-11268G-1), COS7 (ATCC, CRL-1651), and U2OS (ATCC, HTB-96) cells were maintained in DMEM (GIBCO) supplemented with 10% FBS (GIBCO or CellMax) at 37°C with 5% CO_2. Cells were seeded in 6-well plates or 10 cm dishes one day before transfection. Plasmids were transfected using PEI (Polysciences Inc., 23966-2) (Fukumoto et al., 2010) or Lipofectamin 2000 (Invitrogen) following the manufacturers' instructions.

Antibodies

Rabbit anti-GFP polyclonal antibody was self-produced and purified (Wang et al., 2015) (IP (Immunoprecipitation) 1:500; IB (Immunoblotting) 1:5,000). Rabbit anti-Calu1 (ProteinTech, #17804-1-AP. IP 1:200; IB: 1:1000), rabbit anti-Calnexin (Cell Signaling Technology, #2679. IF: 1:200), mouse anti-Climp63 (Enzo, #ALX-804-604. IP 1:500; IB 1:5,000), mouse anti-Flag (Sigma-Aldrich, #F1804. IB 1:2,000), mouse anti-HA (Sigma-Aldrich, #H9658. IP 1:500; IB 1:5,000), mouse anti-GAPDH (CWBIO, #0100A. IB 1:5,000), rabbit anti-Rtn4 (Proteintech, #10740, IB 1:1,000. This antibody recognizes all reticulon proteins and detected Rtn4b (~50 kDa) in COS7 cells in this paper.), mouse anti-α-tubulin (Proteintech, #66031, IF 1:1,000; IB 1:10,000), mouse anti-GFP (abcam, #ab290, IP 1:1,000), and rabbit anti-GFP (MBL, #598, IB 1:1,000) antibodies were used. HRP-conjugated anti-rabbit IgG (H+L) (Jackson ImmunoResearch, #111-035-003. IB: 1:10, 000) and HRP-conjugated anti-mouse IgG (H+L) (Jackson ImmunoResearch, #115-035-003. IB: 1:10,000) secondary antibodies were used.

Vector Construction

Human Calu1 and Calu2 were cloned before (Wang et al., 2012). To construct GFP-Calu1/Calu2, a GFP tag was inserted between amino acids 19 and 20 of Calu1 and Clau2, immediately after the signal peptide. Calu1 mutants used for mapping were cloned into pcDNA3.1(+) vector (Invitrogen). Full-length of human Climp63 was amplified from cDNA of HEK293T cells and cloned into pcDNA3.1(+)-3'HA (with a HA tag inserted at the C-terminus, gift from Dr. Zengfan Jiang at Peking University), pEGFP-N3 (Invitrogen), and p3×FLAG-CMV-14 (Sigma Aldrich) vectors. The Climp63 truncation mutants were amplified from the full-length Climp63 and cloned into the pEGFP-N3 vector. For Climp63-2×Lumen, sequence of Climp63 (132-602) (luminal domain) were amplified and added immediately after full-length Climp63. Sequences coding Kinectin and a short isoform of p180 (represented as p180s here) (Ogawa-Goto et al., 2007) were from Origene and cloned into pcDNA3.1(+)-3'HA vector. The plasmid HA-Atlastin2 was constructed before (Zhou et al., 2019). The ER marker mCherry-ER was constructed previously by adding the signal peptide of Calu1 (amino acids 1-19) to the N-terminus, and the ER retrieval signal KDEL to the C-terminus of mCherry, followed by cloning into the pcDNA3.1(+) vector (Zheng et al., 2018).

Immunofluorescence

For immunofluorescence and fluorescence microscopy, cells 24 h post-transfection were washed

with pre-warmed PBS, and fixed in 4% PFA (powder was dissolved in PBS) at 37°C for 15 min. The cells were then washed for three times with PBS and permeabilized in 0.15% Triton X-100 (in PBS) at room temperature (RT) for 10 min, followed by PBS wash. Immunostaining was carried out by incubation of the cells with blocking buffer (3% BSA in PBS) for 30 min at RT and then with primary antibodies at 4°C overnight. After being washed with PBS three times, the cells were incubated with secondary antibodies. The cells on the glass coverslips were placed in 50% glycerol (in PBS), and the edges of the coverslips were sealed with nail polish. Samples were imaged using different microscope as required (described in the related sections).

Imaging and ER Distribution Quantification

For intensity ratio of perinuclear/peripheral, we transfected cells with indicated plasmids together with ER marker mCherry-KDEL plasmid (or stained with anti-calnexin antibody). The samples were prepared as described in the "Immunofluorescence" section and observed under a Zeiss LSM-710NLO&DuoScan confocal fluorescence microscope equipped with a 63×/1.42NA oil immersion objective lens. Images with mCherry-KDEL expression or calnexin staining were captured by ZEN software (Zeiss). A 3 μm × 3 μm region of both perinuclear (the brightest region around the nucleus of a cell) and the most periphery (random pick) area in the cell was selected, and the relative average fluorescence intensities in both regions were quantified by Image J (NIH).

For analyzing cells with juxtanuclear accumulation of ER, samples were observed under IX71 fluorescence microscope equipped with a 60×/1.42 NA oil immersion objective lens (Olympus). We assigned random file names and had a researcher blinded to all experimental conditions to score the cells. Cells with aberrant bright signal in perinuclear region were considered as "juxtanuclear accumulation of ER" based on manual judgement.

For distribution area of ER sheets to total ER, samples were imaged under a Zeiss LSM-710NLO&DuoScan confocal fluorescence microscope equipped with a 100×/1.42NA oil immersion objective lens, and images were captured by ZEN software (Zeiss). The area of sheets and total ER were quantified using Image J (Zheng et al., 2018).

Electron Microscopy

For electron microscopy, monolayered cells grown on 35 mm petri-dish (with grids and numbers on the bottom, for cells transfected with fluorescent proteins) or coverslips (for non-transfected cells) were washed with pre-warmed 0.1 M PBS, and fixed with fixation buffer (2.5 % glutaraldehyde, 2% freshly prepared PFA in 0.1 M PBS) for 1 h at room temperature, and further fixed overnight at 4°C. The samples (for cells transfected with fluorescent proteins) were observed under an IX71 fluorescence microscope equipped with a 10×/0.30NA immersion lens (Olympus), and images were captured with DP controller software (Olympus). The cells with successful transfection were randomly selected and marked in the images. After washing with 0.1 M PBS for 3 times, samples were further fixed with a mixture of 1.2 % OsO_4 and 1.5% $KFe(CN)_6$ for 30 min, and then stained with uranyle acetate in 25% ethanol for 30 min on ice. After that, samples were dehydrated in order with graded ethanol, incubated in 100% anhydrous cupric sulfate twice, infiltrated with Epon/ethanol (1:1) overnight, and then imbedded with Epon (Eponf12, ODSA, MNA, PMP-30) in vacuum for 3

days. The above samples were incubated in a 65°C-drying oven for 2-3 days. Then the petri-dishes were discarded, and the coverslips (for non-transfected cells) were dissolved with hydrofluoric acid. Under bright field microscope, the cells were compared with previously-captured fluorescent images according to the position in the numbered grids and the cell shape. GFP-positive ones were marked for sectioning. For non-transfected (wild-type) cells, random cells were chosen. After serial sectioning, the specimens were stained with uranyl acetate for 20 min, and lead citrate for 10 min in order before imaging with a transmission electron microscope (JEOL, JEM 1010).

For quantification of the ER sheet luminal width, the area within 1 um outside of the nuclear envelop were randomly imaged. The ER profiles with membrane structure that extended continuously for at least 500 nm was counted as peripheral ER sheet. For each cell, at least 3 points of one ER sheet profile and at least 50 ER profiles per cell were measured using Image J (NIH), and the average ER luminal width was calculated. The total cell numbers are shown in the corresponding figure legends. For luminal width of nuclear envelope profile, about 200-300 envelope profiles from 7-10 cells were quantified.

CRISPR/Cas9-mediated Knockout

For Calu1 knockout, the oligo targeting the splicing site of exon 4 of *CALU* gene (GAAGAGAGCAAGGAAAGGCT) was synthesized and ligated to the gRNA vector (Chang et al., 2013). For Climp63 knockout, gRNA (CGCCGCGCCCGCCATGCCCT) targeting the start codon of Climp63 was constructed into the pX330 vector as described (Cong et al., 2013). Along with Cas9 and pBabe (puromycin-resistant), these vectors were co-transfected in COS7 cells (for Calu1 knockout) or U2OS cells (for Climp63 knockout). Puromycin (Sigma Aldrich, #P8833, 2 μg/mL) was added 24 h post-transfection with fresh culture medium and was removed 48 h later. Single cells were sorted into 96-well plates by flow cytometry (Beckman Coulter), and the clones with no Calu1 or Climp63 protein expression were screened and identified by immunoblotting. The positive candidates were further sequenced to confirm successful knockout.

RNAi

For RNAi, short hairpin RNA (shRNA) were used. Control shRNA: GCCTTCGTTCATTTACTACTA; shCalu1#1: GGAGTTTGATATGAATCAATT; shCalu1#2: ACGTGACTTATGGCACTTATT; shClimp63: AAGGTGCAGTCTTTGCAAGCC (Shibata et al., 2010). All shRNAs were cloned into the pLKO.1 vector (Wang et al., 2015).

Immunoprecipitation and Immunoblotting

For immunoprecipitation, cells were harvested, washed three times with ice-cold PBS, and lysed in lysis buffer (25 mM HEPES, 150 mM KAc, 2 mM $Mg(Ac)_2$, 1% digitonin, pH 7.4) supplemented with protease inhibitor cocktail (Roche, 04693159001). The lysates were centrifuged at 12,000×g for 15 min at 4°C, and the supernatant were mixed with related antibodies and rotated at 4°C overnight, then followed by conjugation with Protein G Sepharose beads (GE Healthcare) at 4°C for 2 h. After washing with the lysis buffer for three times, the beads were boiled for 10 min at 100°C with SDS loading buffer. The immunoprecipitation samples were separated by SDS-PAGE, transferred to

PVDF membranes (Millipore), blocked with blocking buffer (3% BSA in TBST), and probed with primary antibodies at 4°C overnight. The membranes were washed in TBST for three times before incubating with HRP-conjugated secondary antibody at RT for 2 h.

Microtubule Co-sedimentation

For microtubule co-sedimentation assay, HEK293T cells were transfected with Climp63-HA and GFP or GFP-Calu1 for 36 h. The cells were lysed in PIPES buffer (80 mM PIPES, pH6.8, 1 mM $MgCl_2$, 1 mM EGTA, 100 mM NaCl, 1% TritonX-100, supplemented with complete protease inhibitors) for 30 min on ice, and followed by centrifugation twice at 20,000×g for 20 min at 4°C. The supernatant was supplemented with 1 mM GTP and 40 μM Taxol and then incubated at 4°C or 37°C for 30 min before centrifugated at 20,000×g for 30 min at 4°C or 37°C, respectively. The resulted pellet and supernatant were collected and subjected to immunoblotting analysis. The percentage of pellet was quantified using Image J.

Fluorescence Recovery after Photobleaching (FRAP)

For FRAP assay, COS7 cells were transfected with Climp63-mApple and GFP or GFP-Calu1 plasmids. After 24 h, the cells were observed under a Zeiss LSM-710NLO&DuoScan confocal fluorescence microscope equipped with a 100×/1.42NA oil immersion objective lens. Images were acquired at 600 ms interval and a 2 μm diameter circular region was bleached after the fifth frame. Images were then processed by ZEN software (Zeiss) to quantify the average intensities of the bleached regions.

Statistical Analysis

Statistical analysis was performed using GraphPad Prism 7. The definition of mean value, definition of significance, and sample size were indicated in the figures and corresponding figure legends.

Supplemental References

Chang, N., Sun, C., Gao, L., Zhu, D., Xu, X., Zhu, X., Xiong, J.W., and Xi, J.J. (2013). Genome editing with RNA-guided Cas9 nuclease in zebrafish embryos. Cell Res *23*, 465-472.

Cong, L., Ran, F.A., Cox, D., Lin, S., Barretto, R., Habib, N., Hsu, P.D., Wu, X., Jiang, W., Marraffini, L.A.*, et al.* (2013). Multiplex genome engineering using CRISPR/Cas systems. Science *339*, 819-823.

Fukumoto, Y., Obata, Y., Ishibashi, K., Tamura, N., Kikuchi, I., Aoyama, K., Hattori, Y., Tsuda, K., Nakayama, Y., and Yamaguchi, N. (2010). Cost-effective gene transfection by DNA compaction at pH 4.0 using acidified, long shelf-life polyethylenimine. Cytotechnology *62*, 73-82.

Ogawa-Goto, K., Tanaka, K., Ueno, T., Tanaka, K., Kurata, T., Sata, T., and Irie, S. (2007). p180 is involved in the interaction between the endoplasmic reticulum and microtubules through a novel microtubule-binding and bundling domain. Mol Biol Cell *18*, 3741-3751.

Shibata, Y., Shemesh, T., Prinz, W.A., Palazzo, A.F., Kozlov, M.M., and Rapoport, T.A. (2010). Mechanisms determining the morphology of the peripheral ER. Cell *143*, 774-788.

Wang, Q., Feng, H., Zheng, P., Shen, B., Chen, L., Liu, L., Liu, X., Hao, Q., Wang, S., Chen, J.*, et al.* (2012). The intracellular transport and secretion of calumenin-1/2 in living cells. PloS one *7*, e35344.

Wang, Q., Shen, B., Chen, L., Zheng, P., Feng, H., Hao, Q., Liu, X., Liu, L., Xu, S., Chen, J.*, et al.* (2015). Extracellular calumenin suppresses ERK1/2 signaling and cell migration by protecting fibulin-1 from MMP-13-mediated proteolysis. Oncogene *34*, 1006-1018.

Zheng, P., Chen, Q., Tian, X., Qian, N., Chai, P., Liu, B., Hu, J., Blackstone, C., Zhu, D., Teng, J.*, et al.* (2018). DNA damage triggers tubular endoplasmic reticulum extension to promote apoptosis by facilitating ER-mitochondria signaling. Cell Res *28*, 833-854.

Zhou, X., He, Y., Huang, X., Guo, Y., Li, D., and Hu, J. (2019). Reciprocal regulation between lunapark and atlastin facilitates ER three-way junction formation. Protein Cell *10*, 510-525.
